# Oleic acid inhibits lung Na/K-ATPase in mice and induces injury with lipid body formation in leukocytes and eicosanoid production

**DOI:** 10.1186/1476-9255-10-34

**Published:** 2013-10-31

**Authors:** Cassiano Felippe Gonçalves-de-Albuquerque, Patrícia Burth, Adriana Ribeiro Silva, Isabel Matos Medeiros de Moraes, Flora Magno de Jesus Oliveira, Ricardo Erthal Santelli, Aline Soares Freire, Patrícia Torres Bozza, Mauricio Younes-Ibrahim, Hugo Caire de Castro-Faria-Neto, Mauro Velho de Castro-Faria

**Affiliations:** 1 Instituto Oswaldo Cruz, Laboratório de Imunofarmacologia, Fiocruz, Rio de Janeiro, RJ, Brazil; 2 Departamento de Biologia Celular e Molecular, Instituto de Biologia, Universidade Federal Fluminense, Niterói, RJ, Brazil; 3 Departamento de Química Analítica, Universidade Federal do Rio de Janeiro, Rio de Janeiro, RJ, Brazil; 4 Departamento de Medicina Interna, Faculdade de Ciências Médicas, Universidade do Estado do Rio de Janeiro, Rio de Janeiro, RJ, Brazil

**Keywords:** Oleic acid, Na/K-ATPase, Lung, Lipid body, PGE_2_, LTB_4_

## Abstract

**Background:**

Acute respiratory distress syndrome (ARDS) can emerge from certain pathologies, such as sepsis, fat embolism and leptospirosis, in which the levels of unesterified fatty acids are increased in the patient’s plasma. ARDS is characterized by edema formation, and edema resolution occurs mainly due to the pneumocyte Na/K-ATPase activity. As previously described, increased oleic acid (OA) plasma concentrations induce lung injury by interfering with sodium transport. The first aim of this study was to develop a radioactivity-free assay to detect Na,K-ATPase activity *ex vivo* using a model of OA-induced lung injury in mice. We also investigated the relationship between Na/K-ATPase inhibition and OA-induced lung injury using ouabain-induced lung injury as a comparison, because of the well-described effect of ouabain as a Na/K-ATPase inhibitor.

**Methods:**

We developed a Na/K-ATPase assay based on the capture of non-radioactive Rb^+^ ions by mice lung tissue in the absence or presence of ouabain, a specific Na/K-ATPase inhibitor. Rb^+^ incorporation into the lung was measured by inductively coupled plasma-optical emission spectrometry (ICP-OES) after lung tissue mineralization. Na/K-ATPase activity was considered as the difference between Rb^+^ incorporation in the absence and in the presence of ouabain. Bronchoalveolar lavage fluid was collected for lung injury assessment. For this assessment, cell counting, lipid body enumeration and lipid mediator concentrations were measured. Histological analyses were used to determinate lung pathology. Whole body plethysmographic analysis was performed to assay lung function.

**Results:**

The lung Na/K-ATPase activity of mice was completely inhibited by an OA dose of 10 μmol, an effect also obtained with 10^-3^ μmol of ouabain, as demonstrated by the decreased Rb^+^ incorporation in the lungs. The same OA dose induced lung edema and inflammation with cell influx, lipid body formation, and leukotriene B_4_ (LTB_4_) and prostaglandin E_2_ (PGE_2_) production. Ouabain also induced lung inflammation, as detected by histological examinations. As far as we know, this is the first time that ouabain-induced lung injury was shown. Both OA and ouabain induced functional lung pathology in mice simultaneously with inhibition of the lung Na/K-ATPase activity.

**Conclusions:**

We developed a new non-radioactive assay to quantified Na/K-ATPase *in vivo.* OA and ouabain inhibited *in vivo* Na/K-ATPase activity in the lungs and induced lung injury. Our data reinforce the idea that Na/K-ATPase inhibitors may worsen lung injury in specific pathological conditions.

## Background

The first description of adult respiratory distress syndrome (ARDS) appeared in 1967 [1]. A less severe form of ARDS is characterized by increased alveolus permeability [2]; however, according to the Berlin definition, the term ALI is no longer used, and ARDS can be stratified into mild, moderate and severe [3]. Further improvements in the Berlin definition have already been proposed [4]. The initial lesion in ARDS is an increase in the alveolar capillary permeability to plasma proteins, which leads to an interstitial and alveolar edema [3,5]. The resolution of pulmonary edema and of lung inflammation is a relevant factor for ARDS outcome [6]. Fluid management is one of the most important measures that has been shown to impact ARDS, and a dynamic monitoring of the lung fluid balance seems to influence clinical outcomes [7]. The removal of an alveolar edema depends on the vectorial transport of salt and water across the alveolar epithelium, in part through apically located sodium channels (ENaC), followed by its extrusion into the lung interstitium via the basolaterally located Na/K-ATPase [8-10], which in turn drives the passive water flow toward the capillary net through certain transcellular channels, the aquaporins [10]. Thus, the direct epithelial cell injury and/or defects in ion transport inflicted by bacterial and viral pathogens or by oxidative injury lead to a reduction in fluid clearance [11].

The acute inflammatory response in ARDS compromises the integrity of the alveolar-capillary membrane. In this respect, OA can induce lung pathology that is similar to clinical ARDS [12].

In the present work, we developed a Na/K-ATPase assay based on non-radioactive Rb^+^ uptake by mouse lung tissue 30 min after an intravenous (i.v.) injection of OA, in the presence or absence of ouabain. In addition, we used an *in vivo* mouse model of ARDS induced by OA to evaluate edema formation, lung inflammation, and lung pathology that could result from Na/K-ATPase inhibition.

## Methods

### Animals

All experiments were conducted in male Swiss mice (25 – 35 g) obtained from the Oswaldo Cruz Foundation breeding unit. The animals were lodged at 22°C with a 12 h light/dark cycle and free access to food and water. Animal housing conditions and all experimental procedures conformed to institutional regulations and were in accordance with the National Institute of Health guidelines on animal care. The institutional animal welfare committee approved all of the procedures described here under license number 002–08.

### Preparation of oleate solutions

OA (Sigma-Aldrich, St. Louis, MO) was used to prepare a 100 mM tris-oleate solution as described in Gonçalves de Albuquerque, 2012 [13]. Briefly, after weighting and water addition, sodium hydroxide was slowly added until the pH reached 12.0. The mixture was sonicated to complete oleate solubility, and then, the pH was carefully adjusted to 7.6 with dilute Hydrochloric acid*.* The working oleate solutions were prepared by appropriate dilutions of the 100 mM solution with sterile saline (PBS) pH 7.5. The working oleate solutions were tested for the presence of LPS by the limulus amebocyte lysate test (LAL), which was provided by the Instituto Nacional de Controle de Qualidade em Saúde (INCQS)-Fundação Oswaldo Cruz, and showed negative results.

### Intravenous administration of oleate

Intravenous injections were administered into the orbital plexus (inner angle of the eye ball). Each group received 100 μL of tris-oleate solution containing either 2.5, 5.0 or 10.0 μmol of OA per animal. Edema formation and inflammatory parameters were measured several times after the challenge. Control groups received 100 μL of sterile saline (PBS).

### Na/K ATPase assay in mouse lungs based on Rb^+^ incorporation

The mice were divided into 3 groups and anesthetized with isoflurane. Each animal in the first group received 100 μL of a KCl free-Hank’s solution containing 8 μmol RbCl and 2.0, 5.0 or 10.0 μmol of OA. The second group received the same amount of RbCl and 10^-3^ μmol of ouabain per animal. The third group (controls) received only 8 μmol of RbCl. After 30 min, the animals were sacrificed with isoflurane, and their lungs were removed, rinsed in cold PBS and cut into small pieces. After the removal of excess liquid with filter paper, 0.5 g of the lung tissue was transferred to glass tubes for mineralization. After 5 mL of 65% nitric acid was added, the tubes were heated in a digesting plate (model TE 040125 -Tecnal Ind.) until complete digestion. After cooling, the volumes were standardized at 25 mL using distilled water. Mineralized tissue samples were used to quantify the Rb^+^[14]. Rb^+^ was quantified by inductively coupled plasma optical emission spectrometry (ICP-OES) using an Ultima 2 apparatus with Mira Mist Nebulizer and spray chamber (Jobin Yvon, Longjumeau, France). A rubidium nitrate standard (Ultra Scientific, EUA) was used to construct the calibration curve. The results were expressed in μmol of Rb^+^ incorporated per 30 min per gram of tissue.

### Total and differential cell analysis and total protein assay of bronchoalveolar lavage fluid (BALF)

Bronchoalveolar lavage was performed after the trachea was isolated by blunt dissection. A small caliber tube was inserted into the airway. Three volumes of 1.0 mL of PBS were sequentially instilled, gently aspirated and pooled. In every instillation/aspiration cycle, the same volume (1.0 mL) was recovered from each animal. Total leukocyte counts were performed by optical microscopy in Neubauer chambers after diluting BALF samples in Türk solution (2% acetic acid). Differential leukocyte counts were determined in cytocentrifuged smears stained by the May-Grunwald-Giemsa method. The BALF total protein in the supernatant was determined by the Micron BCA Kit method (Pierce) according to the manufacturer’s instructions.

### Lipid body staining and counting

While still moist, the leukocytes on the cytospin slides were fixed in 3.7% formaldehyde in Ca^2^ + −, Mg^2+^ - free HBSS medium (pH 7.4) and stained with 1.5% OsO_4_, as described by Bozza [15]. Lipid bodies were enumerated by light microscopy with oil-immersion objective lens in 50 consecutively scanned leukocytes*.*

### Leukotriene B_4_ (LTB_4_) and Prostaglandin E_2_ (PGE_2_)

LTB_4_ and PGE_2_ in the BALF supernatants were assayed by EIA kits according to the manufacturer’s instructions (Cayman Chemical, Ann Arbor, Mi).

### Morphological studies

We euthanized the animals using a CO_2_ chamber 30 minutes or 24 h after the intravenous injection of OA, ouabain or sterile saline. The lungs were removed and then fixed in 3.7% neutral buffered formalin, embedded in paraffin, sectioned into 4 μm samples and stained with hematoxylin and eosin. Macroscopic photos were taken after the lung had been removed.

### Airway function measurements

These evaluations were performed on unrestrained animals, individually, 24 hours after the challenge by barometric plethysmography with a whole body plethysmograph (WBP, Buxco, Troy, NY), as previously described [16].

### Statistical analysis

The results were expressed as the means ± SEM and were analyzed with One-way Anova followed by the Neuman-Keuls-Student test. Differences were considered significant at p < 0.05.

## Results

**Figure 1:**
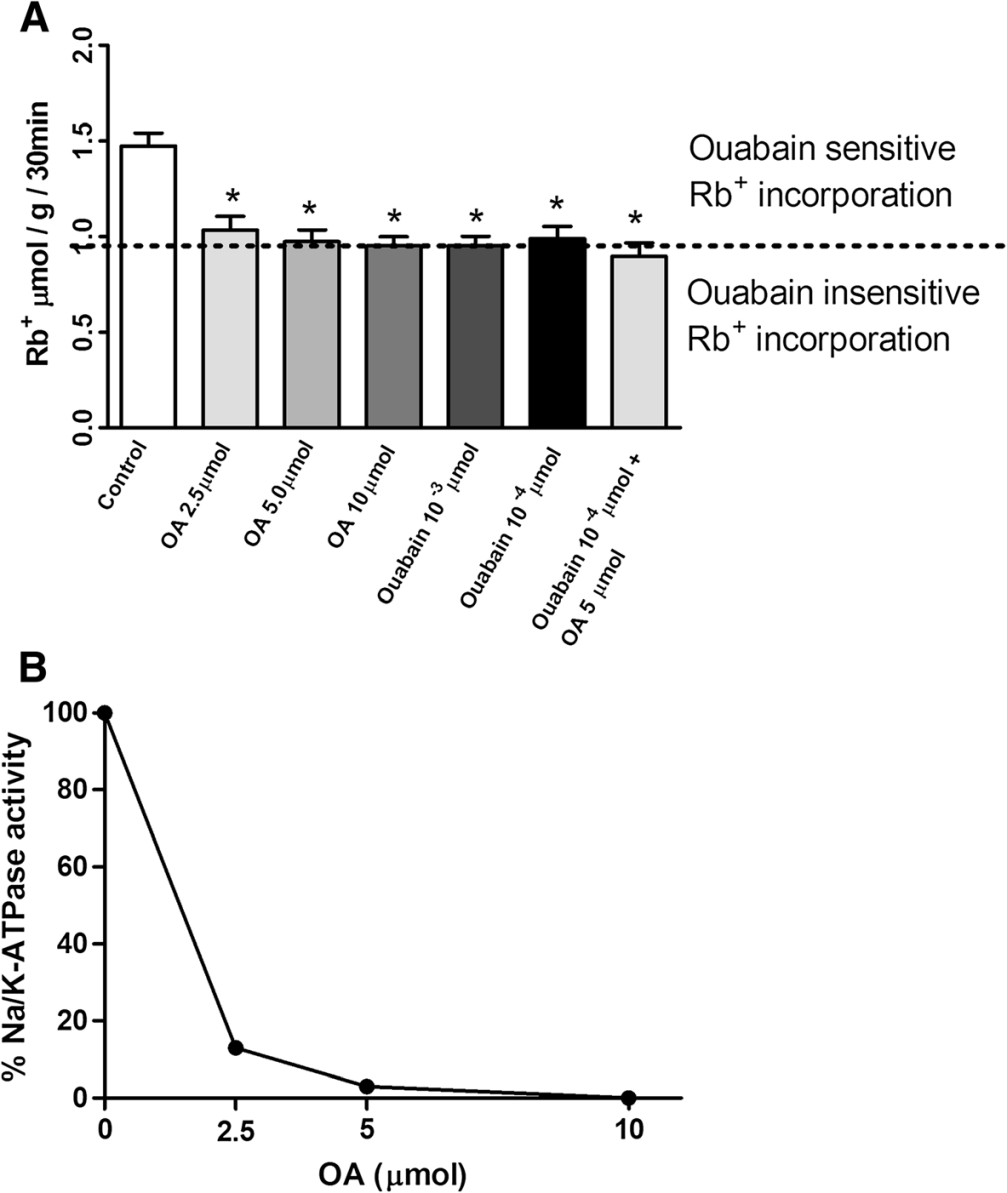
**Inhibition of Rb**^**+ **^**incorporation into lung tissue by OA and ouabain. (A)** Control group was treated solely with 8 μmol Rb^+^. In the experimental groups, 2.5, 5 and 10 μmol of OA; 10^-3^ or 10^-4^ μmol of ouabain; or 10^-4^ μmol ouabain plus OA 5 μmol were injected by the i.v. route together with 8 μmol Rb^+^. Rb^+^ incorporation in lungs was measured after 30 min by ICP-OES in mineralized lung tissues. The results are expressed in μmol Rb^+^ incorporated per h per g of wet tissue ± SEM of 6 to 15 animals in each group. **(B)** Calculated percent inhibition of Na/K-ATPase based on data from Figure 1A (the difference between Rb^+^ incorporation in absence and in presence of ouabain was considered as 100% enzyme activity). *P < 0.0001, compared to controls.

**Figure 2:**
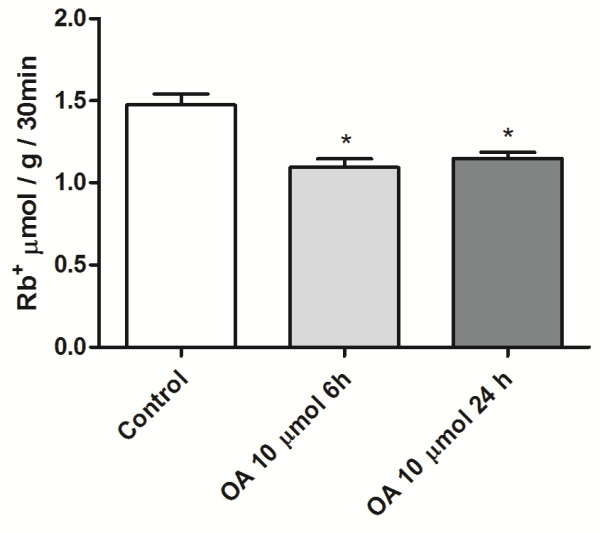
**Inhibition of Rb**^**+ **^**incorporation into lung tissue by OA at 6 and 24 h.** Control group was treated solely with 8 μmol Rb^+^, and 10 μmol of OA was injected by the i.v. route. After 5 h and 30 min or 23 h and 30 min, the animals were injected with 8 μmol Rb^+^. Rb^+^ incorporation in lungs was measured 6 or 24 h after the OA challenge by ICP-OES in mineralized lung tissues. The results are expressed in μmol Rb^+^ incorporated per h per g of wet tissue ± SEM of 6 to 21 animals in each group.

The evaluation of the Na/K-ATPase activity in lung cells was performed by a single injection of Rb^+^ or Rb^+^ plus OA and then measuring the Rb^+^ incorporation into the lung tissue after discounting the basal contamination obtained by inoculating 10^-3^ μmol of ouabain. Figure 1A shows the Rb^+^ incorporation by the lung tissue 30 minutes after its inoculation, as well as the different doses of OA or ouabain or ouabain plus OA. Figure 1B displays the calculated Na/K-ATPase inhibition curve obtained from the data of Figure 1A. We also measured the Rb^+^ incorporation at 6 and 24 h after the challenge (Figure 2). Table 1 shows the percentage of Rb^+^ incorporation after a 10 μmol OA injection. OA inhibition, either of the total amount or ouabain-sensitive Rb^+^ incorporation, was greater at 30 min but remained significant until 24 h

**Table 1:** **Inhibition of the Rb**^**+**^**incorporation in the lung tissue and NKA inhibition by 10 μmol of oleic acid**

	**% of total Rb**^**+**^**uptake and NKA**		
**Parameter analyzed**	**Inhibition by 10 μmol of OA**		
Time	30 min	6 h	24 h
Inhibition of total Rubidium uptake	36%	25.8%	21%
Ouabain-sensitive NKA inhibition^*^	100%	71.8%	63.8%

^*^OA inhibited Rb^+^ incorporation just as ouabain did (10^-3^ μmol) after 30 min.

This was considered to be total NKA inhibition.

**Figure 3:**
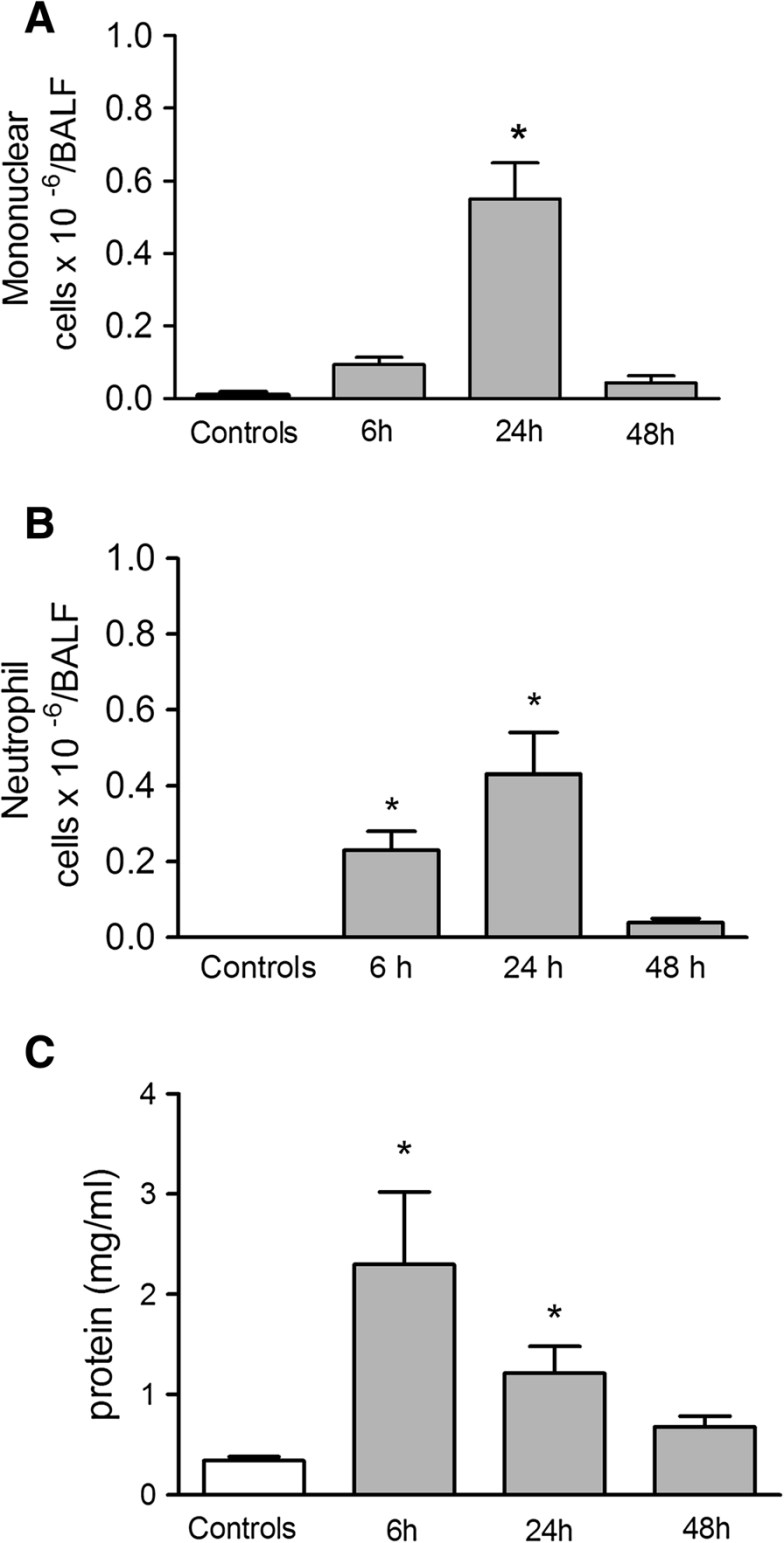
**Cell accumulation and total protein content in BALF 6, 24 and 48 h after OA injection.** Mononuclear cells **(A)**, neutrophils **(B)** and total protein **(C)**. Control group received the same volume of sterile saline. OA (dark columns) was injected i.v. at 10 μmol. The results are means ± SEM from at least 3 different experiments. Each point in an experiment is the mean of 7 different animals. *P < 0.0001, compared to controls.

The onset of ARDS after a 10 μmol OA intravenous injection was characterized by measurements of protein extravasation, leucocyte migration, lipid body formation in leucocytes and PGE_2_ and LTB_4_ production, which were used as markers of lung edema and inflammation in BALF samples. Increased cell migration was detected after 6 hours, but a higher neutrophil infiltration occurred at 24 h after OA administration, returning to basal levels at 48 h (Figures 3A and 3B). Lung edema formation, which was evaluated by assaying the total proteins in the BALF supernatants, occurred as early as 6 h but was less intense at 24 h before returning to basal levels at 48 h (Figure 3C).

**Figure 4:**
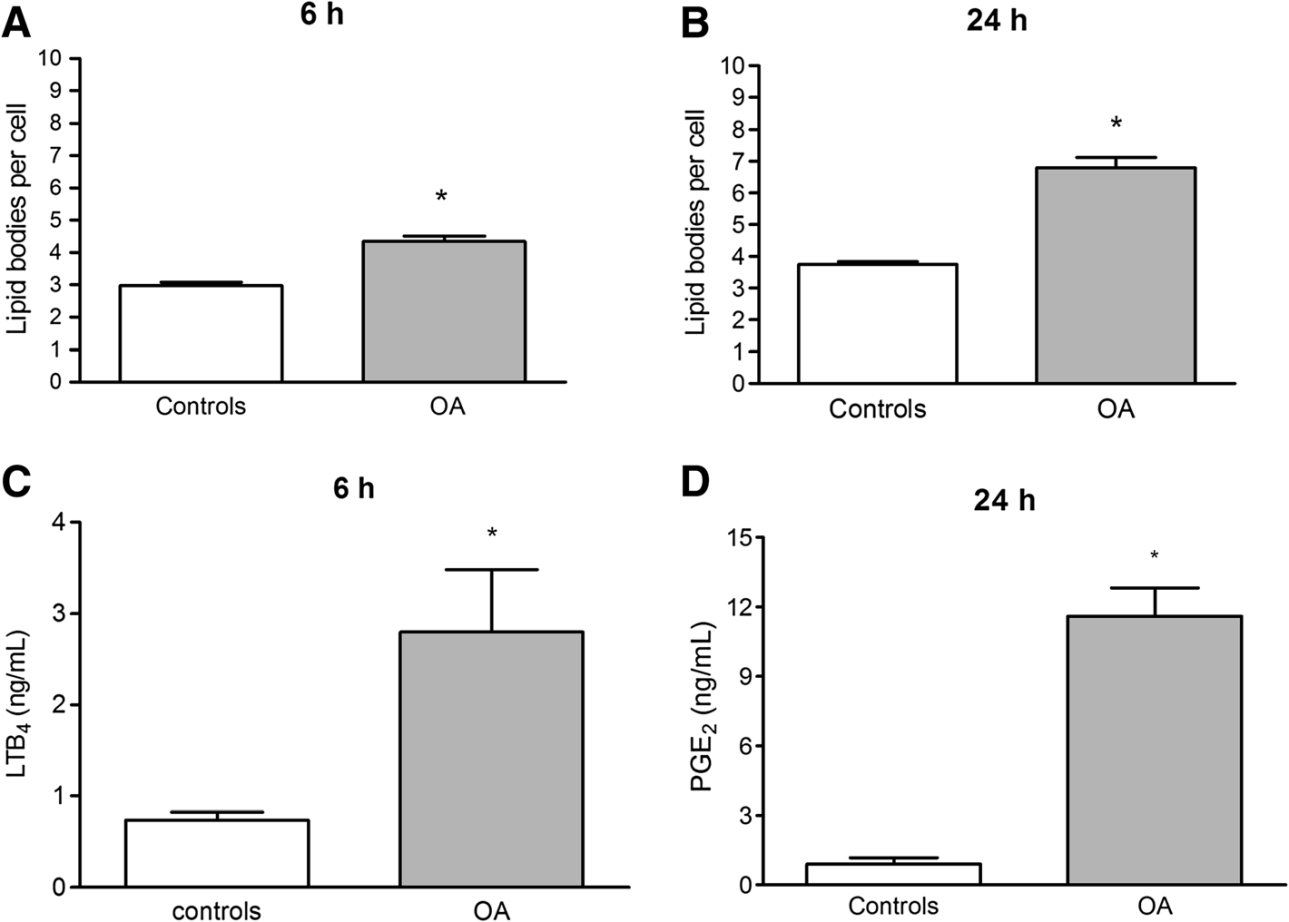
**Lipid body formation in leukocytes and leukotriene B**_**4 **_**and PGE**_**2 **_**production in BALF of OA treated mice.** Animals received 10 μmol OA by i.v. injection (dark columns). Lipid bodies at 6 h **(A)** and 24 h **(B)** after the OA injection were enumerated in 50 consecutive cells in osmium-stained cytocentrifuged smears; LTB_4_**(C)** at 6 h and PGE_2_**(D)** at 24 h after OA challenge were determined in the BALF by ELISA. The control group received sterile saline. The results are means ± SEM of 6 animals in each group. *P < 0.001, compared to controls.

Lipid bodies produce lipid mediators that can be used as markers of cell activation. These markers increased at 6 h and reached a peak at 24 h after the OA treatment (Figure 4A and 4B). The lipid mediator LTB_4_ was elevated at 6 h (Figure 4C), while PGE_2_ was markedly augmented 24 h after OA treatment (Figure 4D).

**Figure 5:**
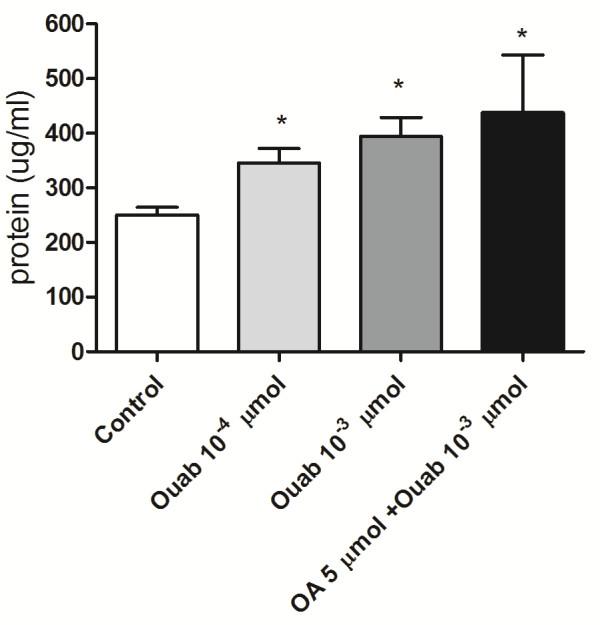
**Total protein in BALF 24 h after ouabain injection.** The control group received the same volume of sterile saline. Ouabain was injected i.v. at 10^-3^ μmol, 10^-4^ μmol or at 10^-3^ μmol plus OA 5 μmol. The results are means ± SEM from at least 6 animals in each group. *P < 0.05, compared to controls.

As edema formation is a characteristic of ARDS, we also measured the protein content in BALF after different doses of ouabain and ouabain plus OA in 24 h (Figure 5). All doses tested caused an increase in the total protein in the BALF, but they did not induce cell accumulation in the BALF at this time point.

**Figure 6:**
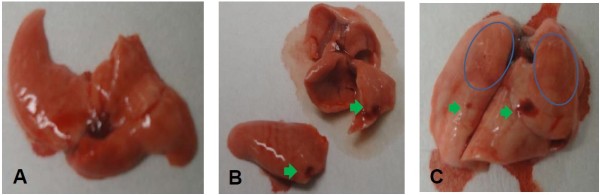
**Illustrative macroscopic examination of lungs after OA or ouabain challenge.** Macroscopic photos of mice lungs challenged with sterile saline **(A)** ouabain 10 ^-3^ μmol/ **(B)**, or OA 10 μmol (**C**). Green arrows point to hemorrhagic points, and blue circles delimitate large hemorrhagic areas.

**Figure 7:**
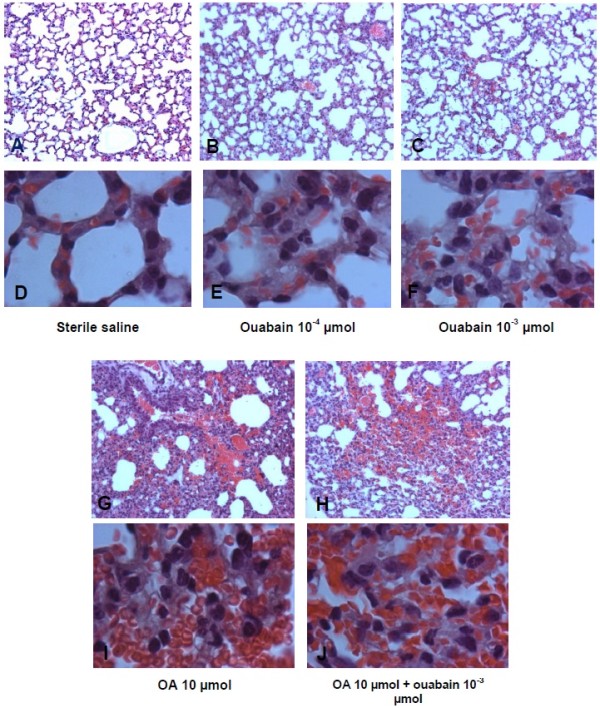
**Illustrative microphotographs of lung pathology caused by OA and ouabain at 30 min.** Sterile saline (control – **A**, **D**); ouabain 10^-4 ^**(B**, **E)**, or 10^-3^ μmol **(C**, **F)**; OA 10 μmol **(G**, **I)** or OA 10 μmol + ouabain 10^-3^ μmol **(H**, **J)** were injected i.v. 30 min before the collection of the lungs. Figures are shown at 100× magnification **(A**, **B**, **C**, **G**, **H)** or 1000× magnification **(D**, **E**, **F**, **I**, **J)**. These pictures are representative of at least 5 animals in each group.

**Figure 8:**
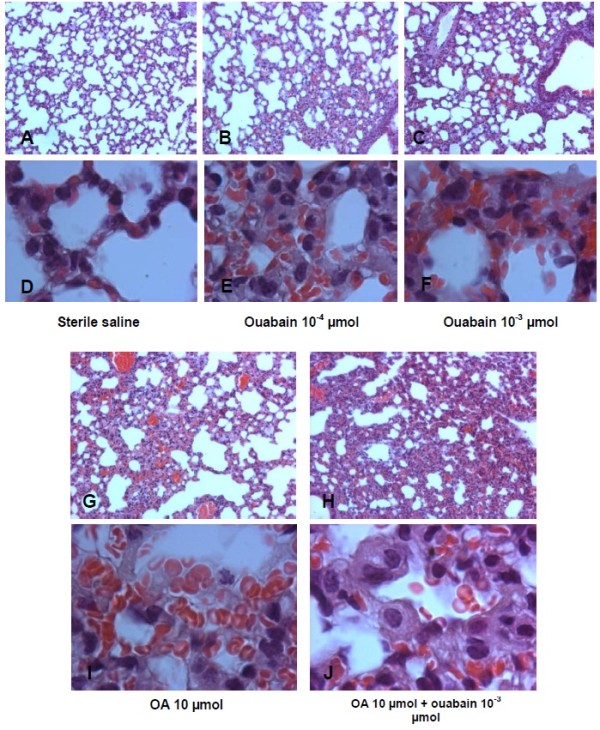
**Illustrative microphotographs of lung pathology caused by OA and ouabain at 24 h.** Sterile saline (control – **A**, **D**); ouabain 10^-4 ^**(B**, **E)**, or 10^-3^ μmol **(C**, **F)**; OA 10 μmol **(G**, **I)** or OA 10 μmol + ouabain 10^-3^ μmol **(H**, **J)** were injected i.v. 24 h before the collection of the lungs. Figures are shown at 100× magnification **(A**, **B**, **C**, **G**, **H)** or 1000× magnification **(D**, **E**, **F**, **I**, **J)**. These pictures are representative of at least 5 animals in each group.

Macroscopic examination of mice lungs injected with ouabain or OA revealed congestion and hemorrhagic areas at 30 min (Figure 6) that were also detected at 24 h (not shown). In addition, we performed histological examinations of the lungs 30 min and 24 h after the challenge with OA, ouabain or ouabain plus OA. To quantify the extent of the lung injury, we adapted a scoring system that takes into account intra-alveolar hemorrhage, leukocyte infiltration and tissue disruption [17]. Table 2 demonstrates that OA and ouabain significantly increased lung injury scores, although the effect was more pronounced with OA. The administration of OA plus ouabain did not have an additive effect because the lung injury scores were similar to the ones observed with OA alone. These results are illustrated in Figures 7 and Figure 8 for the 30 min and 24 h time point analysis, respectively. Hemorrhagic points, leukocytes in the lung tissue and some tissue disruption can be clearly detected after OA or ouabain injury, but, as mentioned above, the effect was more pronounced in the OA-challenged mice. Lung function was also evaluated by whole body plethysmography 30 min after OA or ouabain. Compared to controls, both OA and ouabain mice had significantly increased enhanced breathing pauses (Penh), which was used as an index of airway obstruction (from 0.58 ±0.69 in controls to 1.25 ± 0.34 in ouabain-injected mice and 0.87 ± 0.12 in OA-injected mice).

**Table 2:** Lung injury score extension based on histological analysis

	**Control**	**Ouabain 10**^**-4**^ **μmol**	**Ouabain 10**^**-3**^ **μmol**	**OA**	**OA + Ouabain 10**^**-3**^ **μmol**
*Lung injury score 30 min	-	+	++	+++++	+++++
*Lung injury score 24 h	-	+	+	++++	++++

* Lung injury scores take into account the intra-alveolar hemorrhage, leukocyte infiltration and tissue disruption. The symbol - represents areas without dysfunction and the symbols + to +++++ represent mild to severe lung injury.

## Discussion

The doses of oleic acid inhibited Rb^+^ uptake by the lung tissue, as did the 10^-3^ μmol dose of ouabain, a classical Na/K-ATPase inhibitor. We assume that this ouabain dose completely blocked the rubidium incorporation associated with the Na/K-ATPase activity because higher doses killed the majority of the animals. Lower ouabain doses or ouabain plus OA had similar effects on Rb^+^ tissue incorporation. The somewhat high ouabain-insensitive Rb^+^ measurement can be attributed not only to an incomplete tissue-washing procedure but also to a passive Rb^+^ incorporation through K^+^ channels. The proposed *in vivo* Na/K-ATPase assay has low costs and high reliability and represents an adaptation of the mouse lung assessment method of our previous report, which measured liver Na/K-ATPase activity through a liver perfusion methodology in rats [14].

We chose a model of OA-induced ARDS in mice because the mouse is an extensively used model that remains relevant in the study of lung injury mechanisms. Moreover, inflammation induced by the intravenous administration of OA resembles ARDS in many morphological, histological and physiological aspects [18]. In this regard, ARDS patients or at-risk patients who subsequently develop ARDS have increased plasma OA concentrations [19]. Sepsis patients could develop ARDS [20], as they also present markedly increased plasma OA levels compared to healthy volunteers [21]. Previous work has shown that OA is an endogenous Na/K-ATPase inhibitor [22]. Furthermore, OA was able to inhibit Na/K-ATPase in an ARDS rabbit model, resulting in a significantly increased endothelial permeability [23,24]. In most ARDS patients, the edema resolution and Na,K-ATPase activity are impaired and only patients with reduced edema clearance have a higher mortality [8,25,26] suggesting that Na, K-ATPase is an important player in the pathophysiology of ARDS.

Leukocyte recruitment is essential to the immune response to an injury or an infection [27]. In ARDS, neutrophils are the main cells migrating to lungs [28]. Activated neutrophils release an arsenal of potent molecules and contribute to increased tissue damage and inflammation [29]. Whether neutrophil infiltration is the cause or the consequence of the injury is still debatable because ARDS may develop in patients with neutropenia [5]. We detected an increase in total cell accumulation in the BALF (mainly neutrophils), which was consistent with an ARDS type of reaction induced by OA.

Lipid bodies are cytoplasmic inclusions that are present in different cellular types [30]; they increase in number and size in cells involved in inflammatory and immunologic processes [31]. In our experiments, oleic acid induced lipid body formation in leukocytes, denoting cellular activation [32]. These structures are rich in enzymes and substrates, generating lipid mediators such as LTB_4_ (a potent chemotactic agent to neutrophils) and PGE_2_[15], inducing cell migration and increasing endothelial permeability. It has already been described that OA-induced ALI increased plasma PGE_2_ levels [33]. The crosstalk of intracellular pathways with lipid body formation could stem from OA signaling, including inflammasome activation by the low intracellular concentration of K^+^ due to pump inhibition [34]. However, this activation could also involve the role of this enzyme in other signal transduction pathways [35]. Leukotrienes, including LTB_4_, were not likely to be relevant mediators involved in pathophysiology of acute lung injury induced by oleic acid in pigs [36]. We showed that the increase in BALF LTB_4_ was similar to the increase observed in rats [37]. These observations might indicate the existence of species-specific effects of OA. Importantly, the rise of LTB_4_ and PGE_2_ in human samples preceded ARDS in injured blunt-trauma patients [38]. Similarly, in our model, OA augmented BALF PGE_2_, suggesting that it is suited for comparison with the clinical situation.

Oleic acid-induced Na/K-ATPase inhibition persists until 24 h after the challenge, suggesting that this mechanism may be an important contributor to lung injury. The fact that ouabain-induced lung injury was similar to that induced by OA, but less severe, may indicate that OA has additional mechanisms of injury beyond the inhibition of the Na/K ATPAse. As the Na/K-ATPase is the sole biological target known for ouabain, this observation reinforces the claim that OA may have additional biological targets contributing to lung injury, as discussed above. Nevertheless, lung injury demonstrates a nice correlation with Na/K-ATPase inhibition, both at 30 min and 24 h after the challenge, suggesting a potential causal effect. that oleic acid can cause lung injury with alveoli disruption has been well characterized in animal models [39] and may contribute to the lower Rb^+^ uptake by the lung tissue. Here, the tissue damage was observed macroscopically, and we demonstrated functional repercussions with either ouabain or OA.

As a corollary, patients with high levels of plasma OA have a higher risk of developing ARDS [19] or showing deleterious effects on their heart muscle [40]. Therefore, we can suggest that lowering OA plasma levels could help in the prevention of the high mortality and morbidity of ARDS.

## Conclusion

In conclusion, we developed a new, non-radioactive assay to quantify Na/K-ATPase *in vivo.* The inhibition of the Na/K-ATPase *in vivo* by either OA- or ouabain-induced lung injury in mice. Our data reinforce the idea that Na/K-ATPase inhibition by OA seems to play a relevant role in lung injury during conditions of high plasma OA levels, such sepsis, leptospirosis, pancreatitis and eclampsia.

## Abbreviations

ARDS: Acute respiratory distress syndrome; Na/K-ATPase: Sodium potassium ATPase pump; OA: Oleic acid (18:1n-9); ICP-OES: Coupled plasma-optical emission spectrometry; ENaC: Epithelial apically located sodium channel; LAL: Limulus amebocyte lysate test; BALF: Bronchoalveolar lavage fluid.

## Competing interests

The authors state that they have no conflicts of interest to disclose.

## Authors’ contributions

CFGA – Conception and design of the experiments; participation in the manuscript drafting and direct participation in the experiments. PB – Performance of the Na/K-ATPase experiments based on Rb^+^ incorporation. ARS – Performance of the animal manipulations and aid in drafting the manuscript. IMMM and FMJO – Participation in the animal experiments. PTB – In charge of the lipid bodies and of the design of the lipid mediator experiments. RES and ASF – Performance of data acquisition and analysis of the Na/K-ATPase results on Rb^+^ incorporation. MYI – Participation in the experimental design and in manuscript drafting. HCCFN- Conception of the study, participation in its design and aid in drafting the manuscript. MVCF- Participation in the manuscript drafting and approval of the final version of the manuscript. All authors read and approved the final manuscript.
